# Predicting Malignancy in Transudative Pleural Effusions

**DOI:** 10.3390/jcm15145458

**Published:** 2026-07-12

**Authors:** Celal Satici, Furkan Atasever, Banu Kahriman, Melis Ece Terzioglu, Seda Tural, Sinem Nedime Sokucu

**Affiliations:** Yedikule Chest Diseases and Thoracic Surgery Training and Research Hospital, University of Health Sciences, 34020 Istanbul, Turkey; furkanatasever38@gmail.com (F.A.); banukahriman81@gmail.com (B.K.); melistrzgl@gmail.com (M.E.T.); sedatural@yahoo.com (S.T.); sinemtimur@yahoo.com (S.N.S.)

**Keywords:** transudative pleural effusion, malignant pleural effusion, clinical prediction score

## Abstract

**Objectives**: Although transudative pleural effusions are generally associated with benign conditions, a subset may represent malignant pleural effusions (MPE). Because pleural fluid cytology is often not performed in transudative effusions, malignancy may be underdiagnosed. We aimed to identify predictors of malignancy in transudative pleural effusions and develop a clinical prediction score. **Methods**: We conducted a retrospective cohort study including patients with transudative pleural effusion who underwent pleural fluid cytological analysis between January 2022 and January 2026 at a tertiary chest diseases center. Among 3253 patients who underwent thoracentesis, 314 patients with transudative pleural effusions were included. Clinical, radiological, laboratory, and pleural fluid characteristics were analyzed. Univariable and multivariable logistic regression analyses were performed to identify independent predictors of MPE. A simplified risk score was developed from the final multivariable model and internally validated using bootstrap resampling. **Results**: Among 314 patients, 24 (7.6%) were diagnosed with MPE. In multivariable analysis, pleural pathology on thoracic computed tomography (CT) (OR 7.07, 95% CI 2.61–19.16), parenchymal nodule/mass OR 5.18, 95% CI 1.85–15.54), pleural fluid lactate dehydrogenase (LDH) > 150 U/L (OR 5.09, 95% CI 1.86–13.93), and pleural fluid glucose < 100 mg/dL (OR 3.52, 95% CI 1.12–11.1) were independent predictors of MPE. The 16-point scoring system showed excellent discriminative performance (AUC 0.89). Bootstrap validation confirmed good calibration and model robustness. **Conclusions**: In patients with transudative pleural effusions, pleural pathology on thoracic CT, parenchymal nodule or mass, pleural fluid LDH > 150 U/L, and pleural fluid glucose < 100 mg/dL were independent predictors of MPE. A simple 16-point prediction score derived from these variables demonstrated excellent discriminative performance and may assist in identifying patients who warrant pleural fluid cytological evaluation despite transudative pleural fluid characteristics.

## 1. Introduction

Pleural effusions are commonly classified as transudates or exudates according to Light’s criteria, which compare pleural fluid and serum protein and lactate dehydrogenase (LDH) concentrations [1]. Transudative pleural effusions result primarily from systemic alterations in hydrostatic or oncotic pressure and are most commonly associated with benign conditions such as congestive heart failure, liver cirrhosis, and renal failure [2]. Consequently, transudative pleural effusions are generally considered unlikely to represent malignant pleural effusion (MPE) [3].

However, several reports have documented that a small but clinically important subset of transudative pleural effusions may, in fact, represent MPE [3,4,5,6,7,8,9,10,11,12]. Despite their relatively low prevalence, these cases pose an important diagnostic challenge. Previous studies have reported conflicting findings regarding the role of routine pleural fluid cytology in these patients. While some authors recommend routine cytological examination to avoid missing malignant cases [12], others argue that the relatively low prevalence of malignancy among transudative pleural effusions does not justify routine cytology for all patients [13]. Consequently, some studies have attempted to predict the presence of MPE in transudative pleural effusions, no clear consensus has yet been established regarding the optimal approach to identifying patients who require pleural fluid cytology [9].

Moreover, although several diagnostic models, including the Cancer Ratio, Cancer Ratio Plus, and other laboratory-based prediction models, have been developed to improve the diagnosis of MPE in exudative pleural effusions, these models cannot be directly applied to transudative pleural effusions [14,15]. Therefore, a practical prediction model specifically developed for patients with transudative pleural effusions remains needed.

In line with this, the present study aimed to identify independent predictors of MPE among patients with transudative pleural effusions and to develop a simple clinical prediction score integrating readily available radiological and pleural fluid parameters to guide decisions regarding pleural fluid cytology.

## 2. Materials and Methods

### 2.1. Study Design and Settings

This retrospective cohort study included patients with transudative pleural effusion admitted to Yedikule Chest Diseases and Thoracic Surgery Training and Research Hospital between 1 January 2022 and 1 January 2026. The study was approved by the ethics committee and conducted in accordance with the Declaration of Helsinki.

### 2.2. Study Population

A total of 3253 patients who underwent thoracentesis for pleural effusion at our institution were screened. Of these, 2511 had exudative pleural effusions and 742 had transudative pleural effusions. Among patients with transudative pleural effusions, 428 were excluded because pleural fluid cytological examination was not performed or, in patients with negative pleural fluid cytology and concomitant pulmonary or pleural lesions suspicious for malignancy, pathological confirmation of the lesion was unavailable. Consequently, 314 patients with transudative pleural effusions were included in the final analysis. Inclusion criteria were age ≥ 18 years, transudative pleural effusion, and availability of pleural fluid cytology. Patients with positive pleural fluid cytology were classified as having MPE. In patients with negative pleural fluid cytology but concomitant pulmonary or pleural lesions suspicious for malignancy, pathological confirmation of the lesion was required to establish the final diagnosis. Patients without pathological confirmation of suspicious lesions were excluded to avoid diagnostic misclassification. For patients who underwent repeated thoracentesis, only the biochemical parameters from the first thoracentesis were included in the analysis, whereas the final diagnosis established during follow-up (based on cytology and/or histopathology, when applicable) was used as the reference standard for patient classification.

### 2.3. Data Collection

Demographic data including age, gender, smoking history, and comorbidities were recorded for all patients. Additional data included pleural effusion laterality, pleural effusion size on chest X-ray and thoracic computed tomography (CT; Discovery CT750 HD and LightSpeed VCT scanners, GE Healthcare, Chicago, IL, USA), as well as the presence of loculation or free-flowing effusion, pleural thickening, pleural nodularity, and parenchymal nodules or mass on thoracic CT. Routine blood test parameters such as hemoglobin (Hb), white blood cell count (WBC), neutrophil count, lymphocyte count, and neutrophil-to-lymphocyte (N/L) ratio were documented. Furthermore, pleural fluid parameters obtained via thoracentesis—including pH, lactate dehydrogenase (LDH), total protein, glucose, albumin, and cell count differentials (%)—were recorded. Concurrently obtained blood biochemistry results provided serum levels of LDH, total protein, albumin, glucose, blood urea nitrogen (BUN), creatinine, alanine aminotransferase (ALT), and aspartate aminotransferase (AST). Pleural fluid cytology results and the final diagnosis of the pleural effusion were also documented.

### 2.4. Definitions and Measurements

The differentiation between transudative and exudative pleural effusions was based on Light’s criteria: pleural fluid to serum protein ratio > 0.5, pleural fluid to serum LDH ratio > 0.6, and pleural fluid LDH greater than two-thirds of the upper limit of normal serum LDH (284 IU/L in our laboratory) [1]. In patients receiving diuretic therapy, effusions with a serum-to-pleural fluid albumin gradient greater than 1.2 g/dL were classified as transudates [16].

Cytology from pleural aspiration was categorised as ‘neutrophilic’ in the presence of >50% neutrophil count and as ‘lymphocytic’ when lymphocytes accounted for >50% of the differential cell counts [17].

Pleural effusion laterality was classified as right-sided, left-sided, or bilateral. On chest radiography, pleural effusion size was categorized as minimal when it caused blunting of the costophrenic angle only, moderate when it extended up to the level of the hilum, and massive when it extended beyond the hilum [18]. Pleural effusion size was measured in millimeters based on the anteroposterior depth as assessed on supine axial CT images. Among several measurements taken at the thickest areas of the effusion, the largest measurement was recorded [19].

Thoracic CT images were reviewed for pleural abnormalities. Pleural thickening was categorized as regular (smooth, uniform pleural thickening) or irregular (non-uniform, irregular pleural thickening). Pleural nodularity was defined as the presence of one or more focal nodular pleural lesions, whereas a pleural mass was defined as a discrete pleural-based soft-tissue lesion [20]. Parenchymal lesions were classified as nodules when measuring <3 cm and as masses when measuring ≥3 cm in maximum diameter.

The diagnosis of chronic heart failure (CHF), hepatic hydrothorax, was established based on known criteria [21,22]. MPE was defined by the presence of malignant cells in pleural fluid or pleural biopsy specimens [23].

### 2.5. Statistical Analysis

Data were analyzed using IBM SPSS Statistics (version 27.0; IBM Corp., Armonk, NY, USA) and R software (version 4.5.3; R Foundation for Statistical Computing, Vienna, Austria). Descriptive data were expressed as frequencies and percentages for categorical variables, and as mean ± standard deviation or median (interquartile range) for continuous variables, depending on data distribution. Between-group comparisons were conducted using appropriate statistical tests based on the distribution and type of variables. Predictors of MPE were assessed using univariable logistic regression analysis. Variables with clinical relevance and/or a *p*-value < 0.05 in univariable analysis were included in the multivariable logistic regression model. To reduce the risk of overfitting, the number of variables included in the multivariable model was limited according to the number of outcome events (at least 10 events per variable). Regression coefficients (B), odds ratios (ORs), and 95% confidence intervals (CIs) were reported. Significant independent predictors were categorized based on receiver operating characteristic (ROC) curve analysis, and optimal cut-off values were determined using the Youden index. To enhance clinical applicability, a simplified risk score was developed from the final multivariable model. Regression coefficients were proportionally transformed into integer-based point values, and a composite score was calculated for each patient. The discriminative performance of the scoring system was evaluated using ROC curve analysis, and the optimal threshold was defined according to the Youden index. Diagnostic performance measures, including sensitivity, specificity, positive and negative likelihood ratios, predictive values, and overall accuracy, were calculated at the optimal cut-off point. Internal validation and model stability were assessed using bootstrap resampling with 1000 iterations. Regression coefficients, bias estimates, and 95% confidence intervals were derived from bootstrap samples to evaluate model robustness and potential optimism. Model calibration was evaluated using both statistical and graphical methods. The Hosmer–Lemeshow goodness-of-fit test was used as a supplementary measure. In addition, calibration was assessed by comparing predicted probabilities with observed event rates using a bootstrap-corrected calibration plot based on 1000 resamples. The apparent and bias-corrected calibration curves were plotted against the ideal line. Due to clustering of predicted probabilities in the lower range, the calibration plot was restricted to the 0–0.5 interval to improve visualization.

## 3. Results

A total of 314 patients were included in the study. The median age was 71 years (IQR: 62–79), and 118 patients (37.6%) were female. Active smoking was present in 159 patients (50.6%). Overall, 274 patients (87.3%) had at least one comorbidity, with hypertension (HT) being the most common (*n* = 190, 60.5%), followed by CHF (*n* = 146, 46.5%). Radiological evaluation revealed regular pleural thickening in 51 patients (16.2%), irregular pleural thickening in 2 patients (0.6%), and pleural nodules in 12 patients (3.8%). Parenchymal nodules were observed in 45 patients (14.3%).

Massive pleural effusion was more frequent in the malignant group compared to the benign group (29.2% vs. 13.1%). According to thorax CT findings, absence of pleural pathology was significantly more common in the benign group than in the malignant group (83.1% vs. 33.3%). Regular pleural thickening was also more frequently observed in the benign group (16.9% vs. 8.3%). In contrast, irregular pleural thickening and pleural nodule/mass were observed exclusively in the malignant group. In addition, parenchymal nodule or mass was significantly more frequent in the malignant group compared to the benign group (45.8% vs. 11.7%).

Biochemical analysis demonstrated distinct differences between the groups. Pleural fluid LDH levels were higher in the malignant group compared to the benign group (145 [115–164] vs. 123 [110–143]). In contrast, pleural fluid glucose levels were lower in the malignant group (120 [88–143] vs. 136 [112–177]). Serum LDH levels were lower in the malignant group (241 [196–285] vs. 281 [236–350]), as were serum total protein levels (61.9 [56.5–69.3] vs. 66.1 [59.7–72.1]). The demographic characteristics, radiological findings, pleural fluid analyses, and serum biochemical and hematological parameters of the study population are summarized in Table 1 and Table 2.

Regarding pleural fluid etiology, 24 patients (7.6%) were diagnosed with MPE. Among benign causes, the most frequent etiology was cardiac-related effusion, observed in 157 patients (50%), followed by renal failure in 34 patients (10.8%). In 80 patients (25.5%), the etiology of pleural effusion remained undetermined despite diagnostic evaluation. The distribution of all pleural fluid etiologies is summarized in Table 3.

In the univariable logistic regression analysis performed to identify predictors of MPE, presence of massive pleural effusion, pleural pathology on thoracic imaging, presence of parenchymal nodule or mass, elevated pleural fluid LDH, low pleural fluid glucose level, low serum LDH, and low serum protein level were found to be significantly associated with MPE (*p* < 0.05 for all). Pleural fluid LDH and glucose levels were further evaluated by ROC curve analysis, and optimal cut-off values were determined using the Youden index as 150 U/L and 100 mg/dL, respectively. Clinically more relevant variables with higher effect sizes were included in the multivariable regression analysis. Accordingly, a total of four variables were entered into the final multivariable model. In the multivariable logistic regression analysis, pleural pathology on thorax CT (OR: 7.07, 95% CI: 2.61–19.16, *p* < 0.001), presence of parenchymal nodule or mass (OR: 5.18, 95% CI: 1.85–15.54, *p* = 0.002), pleural fluid LDH > 150 U/L (OR: 5.09, 95% CI: 1.86–13.93, *p* = 0.002), and pleural fluid glucose < 100 mg/dL (OR: 3.52, 95% CI: 1.12–11.1, *p* = 0.032) were identified as independent predictors of MPE (Table 4).

**Figure 1:**
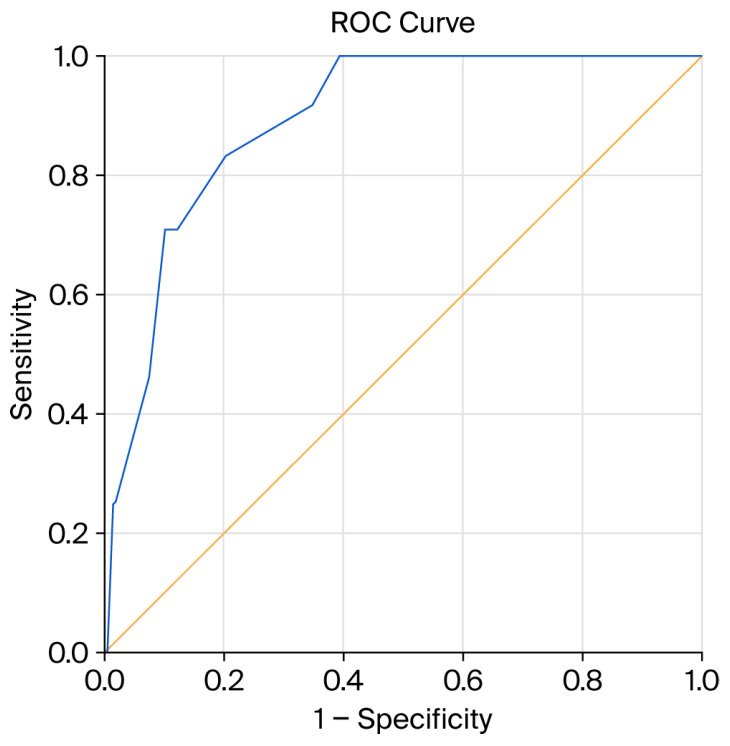
ROC Curve Demonstrating the Discriminative Performance of the Simplified Scoring System for MPE. The blue line represents the ROC curve of the simplified scoring system, while the orange diagonal line represents the line of no discrimination (reference line).

A simplified point-based risk score was derived from the final multivariable logistic regression model by scaling the β coefficients (0.4 log-odds per point) and rounding to the nearest integer. Accordingly, pleural pathology on thorax CT, presence of parenchymal nodule or mass, pleural fluid LDH > 150 U/L, and pleural fluid glucose < 100 mg/dL were assigned 5, 4, 4, and 3 points, respectively (total score range: 0–16). Model calibration was acceptable (Hosmer–Lemeshow *p* > 0.05). Discriminative performance was assessed using ROC curve analysis, yielding an AUC of 0.89 (*p* < 0.001), indicating excellent diagnostic accuracy. The ROC curve of the scoring system is presented in Figure 1. The optimal cut-off value, determined by the Youden index, was ≥8. At this threshold, the model demonstrated high diagnostic performance. Detailed measures of sensitivity, specificity, likelihood ratios, predictive values, and overall accuracy are summarized in Table 5.

To assess the internal validity and stability of the model, bootstrap resampling and calibration analyses were performed. Bootstrap validation (1000 resamples) confirmed the robustness of the multivariable logistic regression model. Pleural pathology (B = 1.956, 95% CI: 0.916–3.346, *p* < 0.001), presence of parenchymal nodule/mass (B = 1.645, 95% CI: 0.561–3.037, *p* = 0.002), and pleural fluid LDH > 150 U/L (B = 1.628, 95% CI: 0.439–3.089, *p* = 0.003) remained statistically significant and stable predictors. Pleural fluid glucose < 100 mg/dL showed borderline statistical significance after bootstrap validation (B = 1.258, 95% CI: −0.434–2.638, *p* = 0.051), with a confidence interval crossing zero, suggesting relatively lower stability compared to other predictors. Overall, bootstrap estimates demonstrated minimal bias and consistent confidence intervals, indicating low overfitting and good internal validity.

**Figure 2:**
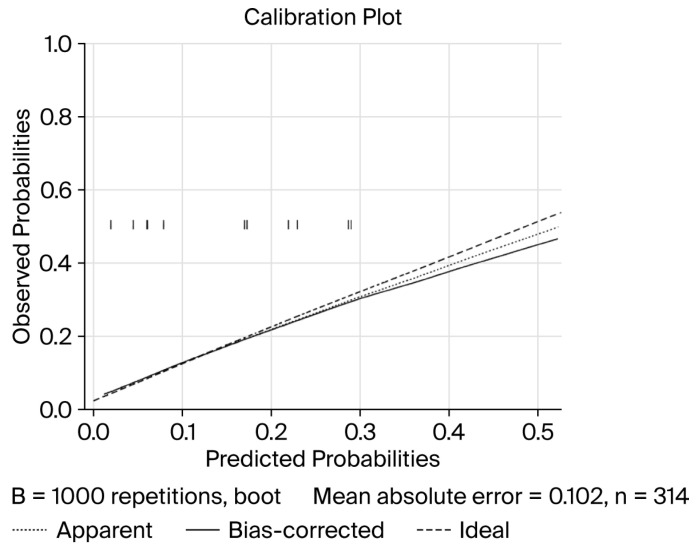
Calibration plot showing the model’s performance across to the risk spectrum.

Calibration analysis demonstrated good agreement between predicted probabilities and observed event rates after bootstrap validation. The bias-corrected calibration curve closely approximated the ideal line, indicating minimal optimism. Minor deviations were observed at higher predicted probability levels, where a slight underestimation of risk was noted. Due to clustering of predicted probabilities in the lower range, the calibration plot was restricted to the 0–0.5 interval to improve visualization (Figure 2). Within this range, the model showed good calibration performance, with close alignment between predicted and observed probabilities across most of the spectrum.

## 4. Discussion

In this study, we challenge the traditional view that transudative pleural effusions are largely non-malignant by demonstrating that malignancy can be accurately predicted using combined clinical and biochemical parameters. Pleural pathology on imaging, parenchymal nodule or mass, elevated pleural fluid LDH, and low pleural fluid glucose emerged as independent predictors. A risk score derived from these variables showed near-perfect discriminatory performance. These findings highlight that malignancy is not negligible in transudative effusions and support a risk-based approach to improve early detection.

Thoracentesis is the initial diagnostic step in the evaluation of pleural effusion, followed by classification into transudates and exudates according to Light’s criteria, which remains the standard approach in clinical practice [17,24]. Transudative effusions generally result from systemic factors leading to altered hydrostatic or oncotic pressures, most commonly in conditions such as heart failure, renal failure, or cirrhosis, and are therefore typically associated with benign etiologies [25]. In contrast, MPE arise from mechanisms such as pleural infiltration by tumor cells, increased vascular permeability, and impaired lymphatic drainage [26]. Consistent with these pathophysiological differences, the majority of MPE demonstrate exudative characteristics, and the likelihood of malignancy is traditionally considered low in transudative effusions [7,13].

In our cohort, 7.6% of patients with transudative pleural effusion were ultimately diagnosed with MPE. This finding is consistent with previous reports indicating that the prevalence of malignancy in transudative effusions ranges between 1% and 10.7% [3,5,7,11,12]. While some studies have suggested that cytological evaluation may be unnecessary in patients with transudative pleural effusion [8], we do not support this approach. Although transudative effusions are commonly associated with benign conditions that can be managed by treating the underlying disease [17], the identification of MPE has important clinical implications, as it necessitates systemic and often palliative treatment strategies [23].

Given that a non-negligible proportion of transudative pleural effusions may be malignant, identifying patients who warrant further diagnostic evaluation with cytological or histopathological sampling is of critical importance. Even when pleural fluid meets transudative criteria, the presence of pleural abnormalities such as thickening, irregularity, or nodularity should raise strong suspicion for malignancy and prompt additional investigation. In our study, the presence of pleural pathology was associated with an approximately sevenfold increase in the risk of malignancy, supporting previous reports in the literature [9]. In addition, the presence of a parenchymal nodule or mass further underscores the importance of cytological evaluation of pleural fluid, as it may indicate underlying malignant involvement, potentially due to micrometastatic disease not detectable by conventional imaging. Based on these findings, we suggest that cytological analysis should be considered in patients with imaging features suspicious for malignancy, even when pleural fluid biochemistry is consistent with a transudate.

In addition to imaging findings, pleural fluid biochemical parameters also provided important diagnostic insights in our study. Pleural fluid LDH levels >150 U/L emerged as an independent predictor, associated with an approximately fivefold increase in the risk of malignancy. Elevated pleural LDH is a well-recognized feature of malignant pleural effusions, reflecting increased cellular turnover and pleural inflammation [26]. Notably, in our cohort, approximately 17% of patients who were classified as having transudative effusions according to Light’s criteria had pleural fluid LDH levels exceeding 150 U/L. This considerable proportion suggests that a subset of transudative effusions may harbor malignant potential.

Pleural fluid glucose represents another important biochemical marker in the evaluation of pleural effusions. Under physiological conditions, pleural fluid glucose levels are comparable to plasma glucose; however, decreased levels may be observed in conditions such as parapneumonic effusions, empyema, and tuberculous pleurisy [25]. Although most malignant pleural effusions do not exhibit markedly reduced glucose levels [27], previous studies have suggested that extensive tumor burden within the pleural space may lead to increased glucose consumption, resulting in lower pleural fluid glucose concentrations [28]. Indeed, pleural fluid glucose levels below 60 mg/dL have been associated with a higher diagnostic yield of cytological examination and poorer prognosis [29]. In our study, pleural fluid glucose levels were lower in the malignant group, supporting the hypothesis of increased metabolic activity of tumor cells within the pleural space. While Ferrerio et al. [9] reported higher glucose levels in non-malignant effusions, our findings suggest a contrasting pattern. Importantly, a pleural fluid glucose level < 100 mg/dL was identified as an independent predictor of malignancy, associated with an approximately 3.5-fold increased risk. Based on these findings, we propose that, in addition to the well-recognized threshold of <60 mg/dL in exudative effusions [29], a pleural fluid glucose level < 100 mg/dL in transudative effusions should also raise suspicion for underlying malignancy.

Several prediction models have been developed to identify MPE in patients with exudative pleural effusions. Among these, the MAPED score incorporates age, pleural effusion size, pleural neutrophil count, pleural protein, and pleural LDH, and demonstrated good diagnostic performance, with an AUC of 0.81 in the derivation cohort and 0.72 in the external validation cohort. Furthermore, it correctly identified 79% of cytology-negative malignant pleural effusions, highlighting its complementary role alongside cytology [30]. In contrast, our model was specifically developed for patients with transudative pleural effusions, a population that has largely been excluded from previous prediction models, and demonstrated excellent discriminative performance with an AUC of 0.89. Similar to MAPED score, our model integrates readily available variables; however, radiological findings—including pleural abnormalities and parenchymal nodules or masses—contributed the greatest predictive value, while pleural fluid LDH and glucose provided additional discriminatory information.

From a clinical perspective, the proposed scoring system offers a practical and easily applicable approach for risk stratification using objective and widely available parameters. Rather than replacing clinical judgment, it is intended to support decision-making by identifying patients with transudative pleural effusions who may warrant closer diagnostic evaluation despite their transudative biochemical profile. In particular, patients with higher scores may benefit from a more comprehensive diagnostic work-up, including repeat pleural fluid cytology, pleural biopsy, or other appropriate investigations when the initial evaluation is nondiagnostic, whereas those with low scores may be managed with greater confidence in the appropriate clinical context. As with other clinical prediction models, the proposed score should be interpreted in conjunction with the overall clinical and radiological assessment rather than as a stand-alone diagnostic tool.

This study has several limitations. First, its retrospective design may have introduced selection and information bias. Second, the study was conducted at a single tertiary center, which may limit the generalizability of the findings. Third, transudative pleural effusions were classified according to Light’s criteria, with the serum-to-pleural fluid albumin gradient applied in patients receiving diuretic therapy. However, the serum-to-pleural fluid protein gradient was not evaluated, and pleural fluid NT-proBNP measurements were not routinely performed; therefore, these parameters could not be incorporated into the present analysis. Another limitation is that pleural effusion size on chest radiography was assessed using posteroanterior chest radiographs only, which may have resulted in less precise estimation of pleural effusion volume compared with additional radiographic views or thoracic ultrasonography. Furthermore, pleural loculation was evaluated using thoracic CT rather than pleural ultrasonography, which is more sensitive for detecting pleural septations and loculations. Despite these limitations, the study has several strengths. Internal validation using bootstrap resampling demonstrated the robustness and stability of the model, suggesting a low risk of overfitting. In addition, the proposed scoring system integrates readily available clinical, radiological, and pleural fluid parameters into a simple and practical tool that may facilitate standardized risk assessment in routine clinical practice. Nevertheless, external validation in independent, prospective, multicenter cohorts is warranted before widespread clinical implementation.

## 5. Conclusions

In conclusion, MPE should be considered even in patients with transudative pleural effusions, particularly when pleural or parenchymal abnormalities are present on imaging. Careful clinical and radiological assessment should guide further diagnostic evaluation. The proposed scoring system may serve as a practical decision-support tool for risk stratification and identifying patients who may benefit from additional diagnostic investigations, but it should always be interpreted in conjunction with clinical judgment rather than as a stand-alone diagnostic tool.

## Figures and Tables

**Table 1:** Demographic and Radiological Characteristics of the Study Population.

Parameters	All Patients *n* = 314	Benign Pleural Effusions *n* = 290 (92.4%)	Malignant Pleural Effusions *n* = 24 (7.6%)	*p* Value
Age, median (IQR)	71 (62–79)	71 (62–79)	70 (62–75)	0.78
Female gender, *n* (%)	118 (37.6)	106 (36.6)	12 (50)	0.19
Active smoking status, *n* (%)	159 (50.6)	146 (50.3)	13 (54.2)	0.72
Pack-years, median (IQR)	40 (25–50)	40 (25–50)	50 (38–68)	0.19
Comorbidities, *n* (%)				
Any comorbidities	274 (87.3)	254 (87.6)	20 (83.3)	0.53
HT	190 (60.5)	178 (61.4)	12 (50)	0.27
CAD	129 (41.1)	122 (42.1)	7 (29.2)	0.22
AF	74 (23.6)	66 (22.8)	8 (33.3)	0.24
DM	94 (29.9)	88 (30.3)	6 (25)	0.58
CHF	146 (46.5)	137 (47.9)	7 (29.2)	0.08
CKD	66 (21)	62 (21.4)	4 (16.7)	0.58
COPD	98 (31.2)	90 (31)	8 (33.3)	0.82
Asthma	13 (4.1)	12 (4.1)	1 (4.2)	0.99
CVD	16 (5.1)	14 (4.8)	2 (8.3)	0.35
Hepatic hydrothorax	5 (1.6)	5 (1.6)	-	1.00
Laterality, ***n*** (%)				0.76
Right	109 (34.7%)	102 (35.2%)	7 (29.2%)	
Left	82 (26.1%)	76 (26.2%)	6 (25%)	
Bilateral	123 (39.2%)	112 (38.6%)	11 (45.8%)	
Pleural effusion size (mm) on thorax CT, median (IQR)	57.5 (40–84.25)	55 (39–81)	78 (46–100)	0.054
Pleural effusion size on chest X-ray, ***n*** (%)				**0.017**
Minimal	120 (38.2%)	115 (39.7%)	5 (20.8%)	
Moderate	149 (47.5%)	137 (47.2%)	12 (50%)	
Massive	45 (14.3%)	38 (13.1%)	7 (29.2%)	
Loculation	49 (15.6%)	44 (15.2%)	5 (20.8%)	0.56
Pleural pathology on thorax CT, ***n*** (%)				**<0.001**
None	249 (79.3%)	241 (83.1%)	8 (33.3%)	
Regular thickening	51 (16.2%)	49 (16.9%)	2 (8.3%)	
Irregular thickening	2 (0.6%)	-	2 (8.3%)	
Nodule or mass	12 (3.8%)	-	12 (50%)	
Parenchymal nodule or mass, ***n*** (%)	45 (14.3%)	34 (11.7%)	11 (45.8%)	**<0.001**

Abbreviations: Hypertension (HT), coronary artery disease (CAD), atrial fibrillation (AF), diabetes mellitus (DM), congestive heart failure (CHF), chronic kidney disease (CKD), chronic obstructive pulmonary disease (COPD), cerebrovascular disease (CVD), computed tomography (CT). Continuous variables are presented as median (interquartile range [IQR]), and categorical variables are presented as *n* (%). Percentages are calculated within each study group unless otherwise. Bold *p* values indicate statistical significance (*p* < 0.05).

**Table 2:** Pleural fluid and Serum Parameters of the Study Population.

Parameters	All Patients *n* = 314	Benign Pleural Effusions *n* = 290 (92.4%)	Malignant Pleural Effusions *n* = 24 (7.6%)	*p* Value
Pleural fluid parameters, median (IQR)				
LDH (IU/L)	123 (110–144)	123 (110–143)	145 (115–164)	**0.033**
Protein (g/L)	27.6 (21.8–33)	27.4 (22–32.9)	31.5 (20.9–35)	0.27
Glucose (mg/dL)	134.5 (111–170)	136 (112–177)	120 (88–143)	**0.003**
Albumin (g/L)	17.6 (13.4–20.8)	17.4 (13.3–20.8)	18.3 (13.6–24.1)	0.37
ADA (U/L)	5.2 (3.7–8)	5.1 (3.6–7.9)	5.6 (4–9.9)	0.52
Cell predominance, ***n*** (%)				0.51
Lymphocytic	305 (97.1%)	282 (97.2%)	23 (95.8%)	
Neutrophilic	9 (2.9%)	8 (2.8%)	1 (4.2%)	
Serum parameters, median (IQR)				
LDH (IU/L)	279 (230–345)	281 (236–350)	241 (196–285)	**0.003**
Protein (g/L)	66 (59–72)	66.1 (59.7–72.1)	61.9 (56.5–69.3)	**0.04**
Glucose (mg/dL)	123 (101–170)	125 (101–173)	116 (92–144)	0.16
Albumin (g/L)	37 (32–41)	37.1 (32.3–40.9)	34.6 (29.1–39.4)	0.14
Hb (g/dL)	11.2 (9.7–12.9)	11.2 (9.7–12.8)	11.7 (9.7–13.8)	0.79
Leukocyte (10^3^/μL)	8.75 (6.72–11.33)	8.71 (6.72–11.37)	9.2 (7.15–11.18)	0.61
N/L Ratio	5.27 (3.2–9.63)	5.23 (3.2–9.32)	9.64 (3.11–13.23)	0.12
BUN (mg/dL)	45 (32–63)	46 (33–64)	38 (29–53)	0.22
Creatinine (mg/dL)	0.95 (0.74–1.25)	0.95 (0.75–1.25)	0.92 (0.63–1.09)	0.22
AST (U/L)	22 (16–31)	22 (16–32)	19 (14–29)	0.36
ALT (U/L)	18 (12–28)	18 (12–27)	15 (10–41)	0.69

Abbreviations: Adenosine deaminase (ADA), lactate dehydrogenase (LDH), hemoglobin (Hb), neutrophil-to-lymphocyte ratio (N/L), blood urea nitrogen (BUN), aspartate aminotransferase (AST), alanine aminotransferase (ALT). Continuous variables are presented as median (interquartile range [IQR]), and categorical variables are presented as *n* (%). Percentages are calculated within each study group unless otherwise specified. Bold *p* values indicate statistical significance (*p* < 0.05).

**Table 3:** Etiology of transudative pleural effusions.

Benign Etiology (*n* = 290)	*n* (%)	Malignant Etiology (*n* = 24)	*n* (%)
Cardiac failure	157 (54.1%)	Lung adenocarcinoma	12 (50%)
Renal failure	34 (11.7%)	Small cell lung carcinoma	3 (12.5%)
Hypoalbuminemia	6 (2.1%)	Gastric cancer	4 (16.8%)
Hepatic hydrothorax	5 (1.7%)	Breast cancer	3 (12.5%)
Pulmonary embolism	4 (1.4%)	Mesothelioma	1 (4.1%)
Chronic pleuritis	8 (2.8%)	Pancreatic cancer	1 (4.1%)
Tuberculosis	1 (0.3%)		
Unknown	75 (25.9%)		

Percentages are calculated within each study group.

**Table 4:** Univariable and Multivariable Logistic Regression Analyses for Predictors of Malignant Pleural Effusion.

	Univariate Analysis			Multivariate Analysis		
	OR	CI (95%)	*p* Value	OR	CI (95%)	*p* Value
Parameters						
Age	1.002	0.87–1.04	0.93			
Female gender	1.74	0.75–4	0.19			
Smoking	1.17	0.51–2.69	0.72			
HT	0.63	0.27–1.45	0.28			
CAD	0.57	0.23–1.41	0.22			
AF	1.7	0.7–4.14	0.25			
DM	0.77	0.29–1.99	0.58			
CHF	0.46	0.19–1.14	0.094			
CKD	0.74	0.24–2.23	0.59			
COPD	1.11	0.46–2.69	0.82			
Asthma	1.01	0.13–8.1	0.99			
CVD	1.79	0.38–8.93	0.46			
Laterality						
Right	1	ref.				
Left	1.15	0.37–3.56	0.81			
Bilateral	1.43	0.54–3.83	0.48			
Pleural effusion size thorax on CT (mm)	1.01	0.99–1.02	0.13			
Pleural effusion size on chest X-ray						
Minimal	1	ref.				
Moderate	2.02	0.69–5.89	0.2			
Massive	4.24	1.27–14.1	**0.019**			
Loculation	1.47	0.52–4.15	0.47			
Pleural pathology on thorax CT	9.84	3.99–24.2	**<0.001**	7.07	2.61–19.16	**<0.001**
Parenchymal nodule or mass	6.37	2.65–15.3	**<0.001**	5.18	1.85–15.54	**0.002**
Pleural fluid LDH > 150 (IU/L)	5.59	2.36–13.2	**<0.001**	5.09	1.86–13.93	**0.002**
Pleural fluid protein (g/L)	1.03	0.98–1.09	0.25			
Pleural fluid glucose < 100 (mg/dL)	4.68	1.84–11.9	**<0.001**	3.52	1.12–11.1	**0.032**
Pleural fluid albumin (g/L)	1.01	0.98–1.04	0.46			
Pleural fluid ADA (U/L)	1.05	0.98–1.16	0.17			
Cell predominance in pleural fluid						
Lymphocytic	1	ref.				
Neutrophilic	1.53	0.18–12.7	0.69			
Serum parameters						
Serum LDH (IU/L)	0.991	0.98–0.99	**0.008**			
Serum protein (g/L)	0.96	0.92–0.99	**0.045**			
Serum glucose (mg/dL)	0.99	0.98–1.01	0.07			
Serum albumin (g/L)	0.95	0.89–1.01	0.09			
Hb (g/dL)	0.98	0.89–1.1	0.84			
Leukocyte (10^3^/μL)	1.001	1.0001–1.01	0.53			
N/L Ratio	1.03	0.99–1.07	0.127			
BUN (mg/dL)	0.99	0.96–1.01	0.39			
Creatinine (mg/dL)	0.58	0.23–1.47	0.25			
AST (U/L)	1.001	0.99–1.01	0.91			
ALT (U/L)	1.002	0.99–1.01	0.7			

Abbreviations: Hypertension (HT), coronary artery disease (CAD), atrial fibrillation (AF), diabetes mellitus (DM), congestive heart failure (CHF), chronic kidney disease (CKD), chronic obstructive pulmonary disease (COPD), cerebrovascular disease (CVD), computed tomography (CT), adenosine deaminase (ADA), lactate dehydrogenase (LDH), hemoglobin (Hb), neutrophil-to-lymphocyte ratio (N/L), blood urea nitrogen (BUN), aspartate aminotransferase (AST), alanine aminotransferase (ALT). Bold *p* values indicate statistical significance (*p* < 0.05).

**Table 5:** Diagnostic Performance of the Simplified Scoring System for Predicting MPE.

Statistic	Value	95% CI
Sensitivity	70.83%	48.91–87.38%
Specificity	90.00%	85.95–93.20%
Positive Likelihood Ratio	7.08	4.61–10.89
Negative Likelihood Ratio	0.32	0.17–0.61
Positive Predictive Value	36.81%	27.48–47.25%
Negative Predictive Value	97.40%	95.26–98.59%
Accuracy	88.54%	84.49–91.85%
AUC	0.89	0.84–0.94
Scoring System: ≥8 vs. <8		

## Data Availability

The datasets generated and/or analyzed during the current study are not publicly available due to patient privacy and ethical restrictions but are available from the corresponding author upon reasonable request.

## Abbreviations

ADA	Adenosine deaminase
AF	Atrial fibrillation
ALT	Alanine aminotransferase
AST	Aspartate aminotransferase
AUC	Area under the curve
BUN	Blood urea nitrogen
CAD	Coronary artery disease
CHF	Congestive heart failure
CI	Confidence interval
CKD	Chronic kidney disease
COPD	Chronic obstructive pulmonary disease
CT	Computed tomography
CVD	Cerebrovascular disease
DM	Diabetes mellitus
Hb	Hemoglobin
HT	Hypertension
IQR	Interquartile range
LDH	Lactate dehydrogenase
MPE	Malignant pleural effusion
N/L	Neutrophil-to-lymphocyte ratio
OR	Odds ratio
ROC	Receiver operating characteristic
SPSS	Statistical Package for the Social Sciences
